# RNA‐binding proteins orchestrating immunity in plants

**DOI:** 10.1111/tpj.70433

**Published:** 2025-09-11

**Authors:** Marcel Bach‐Pages, Athira Menon, Brett Wadley, Alfredo Castello, Gail M. Preston

**Affiliations:** ^1^ Department of Biology University of Oxford South Parks Road Oxford UK; ^2^ Centre for Virus Research MRC‐University of Glasgow Glasgow UK

**Keywords:** RNA‐binding protein, immunity, RNA capping, RNA editing, alternative splicing, polyadenylation, stress granules, P‐bodies

## Abstract

RNA‐binding proteins (RBPs) direct the function and fate of RNA throughout the RNA lifecycle and play important roles in plant immunity, orchestrating the post‐transcriptional reprogramming of the transcriptome following induction of plant immune responses, a process that we term ‘RBP‐mediated immunity’. Although the importance of specific RBPs in plant immunity has been known for many years, this field of research is rapidly expanding as new techniques for global profiling of protein–RNA interactions, together with techniques such as ribosomal profiling and metabolic profiling to monitor mRNA translation and turnover and advanced imaging techniques to study RNA and protein structure and localisation, are uncovering new RBPs and providing new insight into the role of RBPs in plant–microbe interactions. Here we discuss the regulatory roles of RBPs during the RNA lifecycle, with a particular focus on post‐transcriptional processes and how RBP functions alter plants' immunological profile in response to cellular pathogens, drawing both on studies of specific RBPs and insights from global profiling approaches. Unsurprisingly, given their central role in plant immune responses, RBPs can also be targeted by pathogens and therefore represent one of the plant's Achilles' heels. We therefore also review emerging evidence for RBP‐mediated susceptibility in plants. Together, knowledge regarding the regulation, specificity and function of immune‐related RBPs can inform plant‐breeding programmes to generate crops with increased disease resistance.

## Significance Statement

RNA‐binding proteins (RBPs) play a central role in plant immunity by controlling the form, function and fate of RNA and are directly targeted by pathogens to promote susceptibility. This review examines our current understanding of the contribution of RBPs to plant immunity and plant disease development and considers how knowledge of RBP functions and targets can be used to enhance disease resistance.

## INTRODUCTION

RNA‐binding proteins (RBPs) are critical for plant responses to environmental cues by regulating RNAs throughout their life cycle, from genesis to decay (Glisovic et al., 2008; Mateos & Staiger, 2023). An increasing number of studies have highlighted the prominent involvement of RBPs in plant immune responses. However, although recent reviews have discussed the role of immune‐related RBPs in the context of specific aspects of plant RNA biology (e.g. Duarte‐Conde et al., 2022; Godinho et al., 2025; Hao et al., 2021; Hewezi, 2024) one of the last reviews to comprehensively consider the role of RBPs in plant immunity across different stages of the RNA life cycle was authored by Staiger and collaborators in 2013, titled ‘Emerging role for RNA‐based regulation in plant immunity’. This seminal review introduced many of the major mechanisms through which RBPs modulate plant immune responses: the production of protein variants through pre‐RNA splicing; nuclear export; gene silencing by small RNAs and stabilisation (or destabilisation) of mRNA transcripts, together with evidence that certain RBPs are directly targeted by pathogen effectors.

Since this review was published, rapid advances in the technologies available to study RNA biology, including global techniques for analysis of the plant transcriptome, translatome, RNA structure and RNA stability and decay, have transformed our understanding of the post‐transcriptional fate of RNA during plant immune responses, such that the importance of post‐transcriptional processes in plant immunity can no longer be considered to be ‘emerging’ (e.g. Meteignier et al., 2017; Mine et al., 2018; Tang et al., 2023; Thieffry et al., 2022; Xu et al., 2017; Zhang & Ding, 2021). These advances have been complemented by the development of new techniques to study the interactions of proteins and RNA during plant immune responses, such as plant RNA interactome capture (RIC), orthogonal organic phase separation (OOPS) and UV crosslinking and immunoprecipitation (iCLIP), which have uncovered hitherto unsuspected RNA‐protein interactions and allowed us to map protein–RNA interactions to single nucleotide resolution (Bach‐Pages et al., 2020; Curtis & Jeffery, 2021; Le et al., 2022; Lewinski et al., 2024; Liu et al., 2020).

Additional aspects of RNA biology such as RNA editing and methylation, the sequestration of proteins and RNA in condensates such as processing bodies (P‐bodies) and stress granules (SGs), and the secretion of extracellular RNA have been shown to influence the outcome of plant–microbe interactions (e.g. Borniego & Innes, 2023; Chantarachot & Bailey‐Serres, 2018; Chen et al., 2024; Furci et al., 2024; García‐Andrade et al., 2013; Lu et al., 2024; Yang et al., 2020; Yu et al., 2019). Furthermore, as researchers continue to develop a more comprehensive picture of the pathogen ‘effectorome’ (factors secreted by pathogens that influence plant processes), they continue to find new effectors capable of directly or indirectly manipulating RBP function in diverse pathogens (e.g. Huang et al., 2024; Li et al., 2024; Li & Kou, 2025; Wang et al., 2022; Wang, et al., 2022).

It is therefore timely to review our understanding of the role of RBPs in plant immunity. This study reviews current evidence for what we term ‘RBP‐mediated immunity in plants’, in which the activity of RBPs results in cellular reprogramming to generate responses that limit infection. We also highlight the increasing number of RBPs that have been shown to be directly or indirectly subverted by pathogens to promote ‘RBP‐mediated susceptibility’ (Box 1).

Box 1 **Bullet point summary**

- Induction of plant immune responses leads to reprogramming of the plant transcriptome, including post‐transcriptional regulation by RNA‐binding proteins (RBPs).
- RBPs form dynamic interactions with their target RNAs, forming ribonucleoprotein complexes that are central to processes governing the RNA lifecycle.
- Proteome‐wide approaches such as RNA interactome capture have enabled the comprehensive identification of RBPs based on their interaction with RNA *in vivo*, including RBPs lacking canonical RNA‐binding domains.
- RBPs have been linked to post‐transcriptional regulation of plant immunity at all stages of the RNA lifecycle, from capping to splicing, editing, transport and sequestration or degradation.
- RBPs are directly targeted by pathogen effectors to modulate or suppress host physiology and immune responses.

## TRANSCRIPTIONAL REPROGRAMMING IS CENTRAL TO PLANT IMMUNE RESPONSES

In order to examine the role of RBPs in plant immunity, we need to consider the central role of transcriptional reprogramming in plant immune responses. Plants can perceive the presence of a wide range of pathogens using pattern recognition receptors (PRRs) to recognise conserved pathogen‐derived features termed pathogen/microbe‐associated molecular patterns (PAMPs/MAMPs) or plant‐derived molecules that are released during pathogen invasion termed damage‐associated molecular patterns (DAMPs) (Zipfel, 2014). PRR activation elicits a signalling cascade that involves mitogen‐activated protein kinases (MAPKs) and calcium‐dependent protein kinases and results in a defence response referred to as PAMP‐Triggered Immunity (PTI), which includes transcriptional upregulation of defence mechanisms, including pathogenesis‐related (PR) proteins and localised production of antimicrobial compounds (Bjornson et al., 2021; DeFalco & Zipfel, 2021; Lewis et al., 2015; Zipfel, 2014). Thieffry et al. (2022) report that elicitation of PTI leads to temporal waves of transcriptional activity, including the use of alternative start sites (TSSs) and rapid induction of transcription factors that further remodel gene expression.

Some pathogens have evolved proteins and small molecules (generally referred to as effectors) that interfere with PTI (reviewed in Toruño et al., 2016). For example, some bacterial effector proteins impede PAMP detection by promoting degradation of host PRRs, by inhibiting PPR kinase activity and by targeting co‐receptors (Macho & Zipfel, 2015) and nuclear processes (Motion et al., 2015). In addition, effectors can also target regulators of host immunity (susceptibility factors) or act directly as transcription factors to promote disease, all of which directly or indirectly suppress or modulate PTI and damage‐induced transcriptional reprogramming (Perez‐Quintero & Szurek, 2019; Xiang et al., 2025).

Plants possess intracellular receptors that directly or indirectly recognise these effectors and can thereby trigger the induction of an additional layer of defence termed Effector‐Triggered Immunity (ETI), which is commonly associated with a localised programmed cell death response known as the hypersensitive response (HR), which requires transcription of ETI‐associated genes and which influences and is influenced by co‐induction of PTI (Chiang & Coaker, 2015; Lu & Tsuda, 2021; Mine et al., 2018; Ngou et al., 2021; Tian et al., 2021; Yuan et al., 2021).

In addition to local responses, PTI, ETI and pathogen‐associated damage and cell death can result in systemic activation and local and systemic priming of plant defences, and even activation and priming of plant defences in adjacent plants, encapsulated by the general term ‘induced resistance’ (Conrath et al., 2015; De Kesel et al., 2021; Kim & Lim, 2023; Vlot et al., 2021; Wenig et al., 2019). Long‐distance immune signalling in plants has been linked to the activity of numerous mobile and volatile signals, with systemic acquired resistance, a phenomenon in which localised infection or immune challenge leads to enhanced resistance and immune priming in distal tissues, associated with signalling molecules such as salicylic acid (SA), methyl salicylate, jasmonate (JA), pipecolic acid, N‐hydroxypipecolic acid, nitric oxide, reactive oxygen species (ROS), azelaic acid and glycerol‐3‐phosphate (Kim & Lim, 2023; Li et al., 2023). A second form of induced resistance is induced systemic resistance, in which the interaction of plants with plant growth‐promoting microbes in the rhizosphere primes plants to display an enhanced response to subsequent infection, through signalling processes that include JA and ET signalling pathways (Vlot et al., 2021). Many of these signalling molecules are also involved in a wider variety of local and systemic signalling processes associated with biotic and abiotic stress and plant development, with JA and its derivatives being particularly associated with responses to cell damage caused by herbivores and necrotrophic pathogens (Jiang et al., 2023; Kim & Lim, 2023; Parker et al., 2022; Pieterse et al., 2014; Ruan et al., 2019).

Thus, PTI, ETI and induced resistance all involve extensive reprogramming of the plant transcriptome, mediated by transcription factors and by modification of DNA and histones, including modulation of DNA methylation by small RNAs. Upon induction, defence‐related genes are transcribed in the nucleus to establish a defence response against pathogens (Buscaill & Rivas, 2014). However, immune activation also typically leads to a pervasive downregulation of other sets of genes, particularly photosynthetic and chloroplastic genes (Bilgin et al., 2010).

Changes in gene expression during induced resistance can be divided into two categories: specific changes in gene expression in local and systemic tissues, and enhanced gene expression in primed plants upon subsequent pathogen challenge (Baum et al., 2019; Mauch‐Mani et al., 2017). However, reprogramming of the plant transcriptome can also occur at other levels and in different cellular compartments, from post‐transcriptional regulation to translation and RNA degradation (Staiger et al., 2013; Aerts et al., 2022; Duarte‐Conde et al., 2022; Small et al., 2023; Chen et al., 2024). RBPs play a central role in these processes by determining the function and fate of cellular mRNAs.

## RBP‐MEDIATED IMMUNITY IN PLANTS

**Figure 1:**
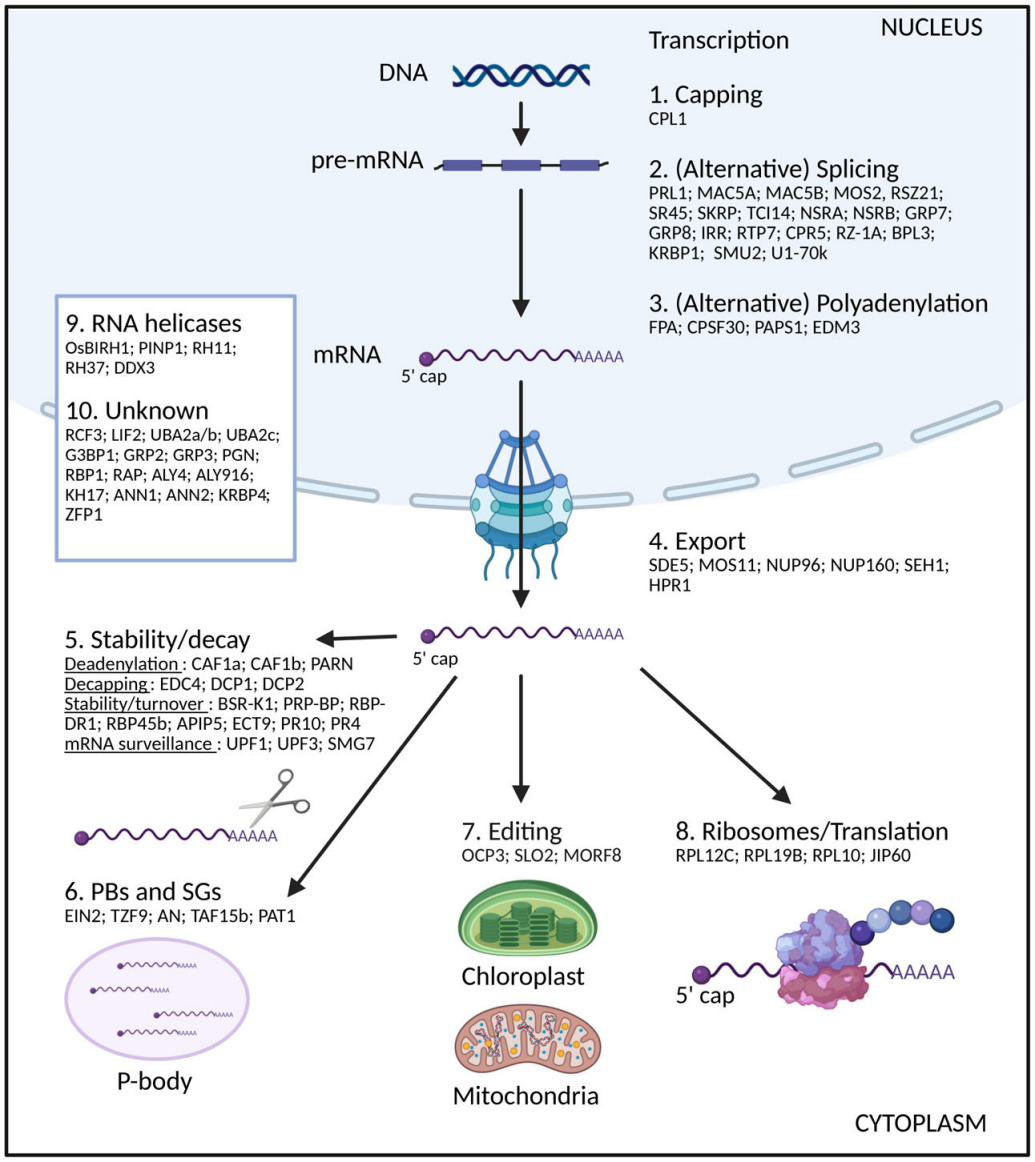
Overview of RNA‐binding proteins (RBPs) involved in plant immunity. RBPs involved in immunity play important roles in all the steps of mRNA metabolism and localisation including transcription, [1] capping, [2] (alternative) splicing, [3] (alternative) polyadenylation, [4] mRNA export to the cytoplasm, [5] mRNA stability/decay, [6] localization to P‐bodies (PBs) or stress granules (SGs), [7] RNA editing, mainly in organelles or [8] ribosomes and translation. [9] RNA helicases are involved in multiple processes in different cellular compartments. [10] Additional RBPs have known roles in immunity, but their precise role in mRNA metabolism and/or localisation awaits discovery. Created with BioRender.com.

RBPs interact with their target RNAs to form ribonucleoprotein (RNP) complexes that are central in processes governing the RNA lifecycle (Glisovic et al., 2008). RNAs are mainly transcribed in the nucleus, and before being translated into proteins, they undergo different processes including capping, splicing and alternative splicing (AS), 3′ end definition, polyadenylation, editing, methylation, nuclear export, cytoplasmic transport and storage. The composition and activity of RNP complexes are highly dynamic, coordinating post‐transcriptional changes according to different developmental processes or in response to external cues. Numerous studies have provided evidence of RBPs playing important roles in plant immunity (Figure 1) and representative RBPs with links to immunity are summarised in Table S1.

RBPs are involved in the transcription of RNA from DNA and in epigenetic regulation of transcription (Parker et al., 2022; Xiao et al., 2019). However, here we will focus on RBPs that interact with RNAs post‐transcriptionally, as the role of the transcription process in plant immunity has been covered in detail elsewhere (Aerts et al., 2022; Buscaill & Rivas, 2014; Li et al., 2016; Tsuda & Somssich, 2015). There is also strong evidence that post‐transcriptional control and the RBPs participating in it play critical roles in viral infection and immunity (Huh & Paek, 2013; Musidlak et al., 2017). This is not surprising since RNA is central in the viral life cycle; however, the role of RBPs in viral infection and immunity is beyond the scope of this review. Here we focus on the post‐transcriptional processes regulated by RBPs that regulate immune responses against cellular pathogens such as bacteria, fungi and oomycetes.

The majority of existing literature on the role of RBPs in plant immunity focuses on RBPs that have classical RNA‐binding domains (RBDs) and well‐characterised molecular functions in RNA biology. However, in recent years, proteome‐wide approaches, such as RIC, have enabled the comprehensive identification of RBPs in cells, tissues and organisms based on their interaction with RNA, offering an unprecedented opportunity to investigate the responses of the RBPome to physiological, environmental and pathological cues (Bach‐Pages et al., 2017; Hentze et al., 2018).

Intriguingly, techniques such as RIC have identified RNA‐binding capabilities in proteins that were not previously predicted or known to interact with RNA (Hentze et al., 2018). While the functional significance of these interactions and their role in plant immunity largely await further investigation, it is important to bear in mind that the diverse protein–RNA interactions discussed below are likely to only represent a fraction of the interactions that occur within plant cells during plant–pathogen interactions. Furthermore, while we introduce and categorise RBPs in terms of relatively well‐established processes within the mRNA lifecycle, ongoing work is drawing attention to an increasing diversity of functions for RBPs and their cognate RNAs at the plant–pathogen interface, particularly in areas such as extracellular vesicles and signalling processes (Manavella et al., 2023; Ruf et al., 2022).

### RNA capping

One of the first steps in the RNA lifecycle is the co‐transcriptional addition of a 7‐methylguanosine (m^7^G) cap at the 5′ end of the pre‐mRNA. This increases RNA stability and translational efficiency and mediates functions such as RNA processing or nuclear export (Ramanathan et al., 2016). RNA POLYMERASE II CARBOXYL TERMINAL DOMAIN (CTD) PHOSPHATASE‐LIKE1 (CPL1) is involved in capping and has recently been linked to immunity. CPL1 contains two double‐stranded RNA (dsRNA) binding domains and a phosphatase domain and participates in the dephosphorylation of the CTD of RNA polymerase II; the mRNA capping process; pre‐mRNA splicing and RNA decay (Bang et al., 2008; Chen et al., 2013; Cui et al., 2016; Jeong et al., 2013; Jiang et al., 2013; Manavella et al., 2012).

CPL1 has been found to affect plant immunity via suppression of PTI‐induced MPK3/MPK6 phosphorylation, ROS bursts and PTI‐induced gene expression (Wei et al., 2023). *cpl1* mutants were observed to be more resistant to the fungi *Fusarium oxysporum* and *Alternaria brassicicola* and the aphid *Myzus persicae*, but not to the bacterial pathogen *Pseudomonas syringae* pv. tomato DC3000 (Thatcher et al., 2018; Wei et al., 2023). However, *cpl1* mutants did show enhanced resistance to *P. syringae* pv. tomato DC3000 *ΔavrPtoΔavrPtoB* when primed with the bacterial immune elicitor flg22 (Wei et al., 2023). This suggests that the effect of CPL1 on immunity could in part be attributed to the role of CPL1 in transcriptional regulation and RNA processing. However, intriguingly, Wei et al. (2023) also reported that CPL1 directly disrupts the interaction of the MAPK enzymes MKK4/MKK5 and MPK3/MPK6 in the nucleus through a mechanism that is dependent on the CPL1 RBD. The exact influence of either mechanism in the immune response remains to be determined.

### RNA splicing

Most eukaryotic pre‐mRNAs contain non‐coding introns that are removed by the spliceosome in a process known as splicing. The spliceosome is a large molecular complex composed of five small nuclear RNPs (snRNPs) and multiple spliceosome‐associated proteins (Plaschka et al., 2018). In a normal splicing event, splice sites located at the 5′ and 3′ ends, together with the branch point within introns, are recognised by snRNPs, enabling the removal of the intronic sequence. However, the recognition of a splice site can be suppressed or enhanced by the activity of trans‐acting regulatory proteins to give rise to alternative splice sites that yield multiple mRNA isoforms from a single pre‐mRNA. This differential splicing process is referred to as AS (Chaudhary et al., 2019). AS expands the complexity of the proteome by affecting multi‐exonic genes in *Arabidopsis thaliana* (Laloum et al., 2018; Marquez et al., 2012). The production of alternative RNA isoforms through AS is regulated in response to environmental cues, particularly cellular stresses and pathogenesis (Gulledge et al., 2012; Martín et al., 2021; Palusa et al., 2007; Tanabe et al., 2007). Over 500 gene transcripts were reported to display flg22‐responsive changes in AS in *A. thaliana* (Bazin et al., 2020), demonstrating that splicing is dynamically regulated during immune responses to pathogens.

Many spliceosome and splicing‐associated factors have been linked to immune‐related functions, highlighting the importance of splicing regulation in plant defences (Godinho et al., 2025; Hewezi, 2024). For example, 16 RBPs involved in splicing showed differential interactions with RNA upon flg22 treatment. Interestingly, the RNA‐binding activity of nuclear splicing factors was found to be stimulated early upon flg22 treatment, while chloroplastic splicing factors were inhibited (Bach‐Pages, Chen, et al., 2020). Components of the MOS4‐ASSOCIATED COMPLEX (MAC), a conserved complex associated with the spliceosome in *A. thaliana*, have been linked to both immunity and development (Johnson et al., 2011). Of these components, MAC5A and MAC5B contain both an RNA recognition motif (RRM) and a CCCH‐type zinc‐finger motif, which have also been linked to RNA‐binding activity and have been confirmed to bind to mRNA and to be required for processing and stability of primary microRNA transcripts (Bach‐Pages, Chen, et al., 2020; Li et al., 2020; Monaghan et al., 2010). MAC5A and MAC5B show partial redundancy, with a double mutant resulting in lethality. A *mac5a‐1* mutant partially suppresses the autoimmune phenotype of the gain‐of‐function mutant *snc1* (SUPPRESSOR OF NPR1‐1, CONSTITUTIVE1 [SNC1]) supporting a role for these RBPs and the MOS4 complex in regulation of plant immunity (Monaghan et al., 2010).

AS can be modulated by the interaction of trans‐acting splicing factors with cis‐regulatory elements in pre‐mRNA. These auxiliary splicing factors are generally categorised into two classes termed Serine–Arginine‐rich (SR) proteins and heterogeneous nuclear ribonuclear‐like (hnRNP) proteins (Meyer et al., 2015). CONSTITUTIVE EXPRESSOR OF PATHOGENESIS‐RELATED GENES 5 (CPR5), a negative regulator of ETI‐associated cell death, belongs to the Transformer 2 subclass of SR splicing factors and has been confirmed to directly bind RNA via its RRM domain (Bowling et al., 1997; Peng et al., 2022). Through a suppressor screen of the *cpr5* mutant, Peng and colleagues found that CPR5 acts upstream of MAC and CLEAVAGE AND POLYADENYLATION SPECIFICITY FACTOR to regulate pre‐mRNA processing within nuclear speckles, which are nuclear domains that are enriched in pre‐mRNA splicing factors (Peng et al., 2022). CPR5 directly interacts with transcripts that are differentially spliced in the *cpr5* loss‐of‐function mutant such as Argonaute RISC component 1 (*AGO1*) and *SR34* (which also belongs to the SR family), which have both been implicated in immune responses (Li et al., 2010; Rigo et al., 2020). Therefore, it was suggested that CPR5 regulates the immune response in *A. thaliana* through direct and indirect effects on AS (Peng et al., 2022).

The impact of SR protein‐mediated modulation of AS on immunity varies between species and pathogens. SR45, which associates with the core spliceosome components U1‐70K and U2AF53, was found to negatively regulate immunity to biotrophic pathogens such as *P. syringae* (Day et al., 2012; Zhang et al., 2017). However, in the wild cotton species *Gossypium australe*, GauSR45a was found to enhance resistance to *Verticillium dahliae*, as silencing GauSR45a reduced the splicing rate of *Verticillium*‐induced immune genes (Liu et al., 2024). Interestingly, the same study reported that different *Gossypium* species showed distinct differences in the number of splicing events induced during infection, with *Verticillium* inducing more AS events in the resistant tetraploid cotton species *Gossypium barbadense* compared to the susceptible tetraploid cotton species *Gossypium hirsutum*.

**Figure 2:**
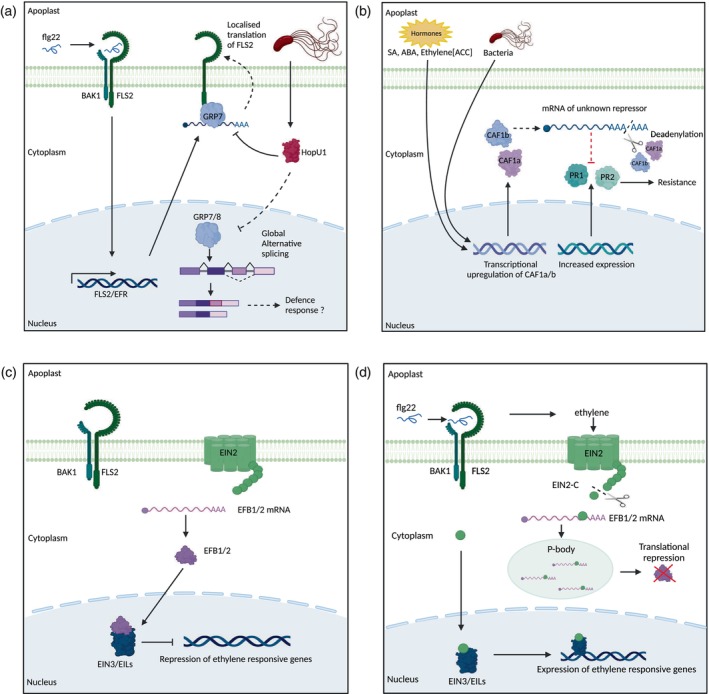
Examples of RNA‐binding proteins with known roles in plant immunity. (a) GRP7 associates with *FLS2* and *EFR* transcripts and is targeted by the effector HopU1. Recognition of the elicitor peptide flg22 by the FLS2‐BAK1 receptor/co‐receptor complex leads to the transcriptional upregulation of the pattern recognition receptor FLS2 and EFR. GRP7 associates with the *FLS2* and *EFR* transcripts and the FLS2 and EFR receptor proteins. These interactions were speculated to result in the localised translation of ligand‐free FLS2 at the plasma membrane. HopU1, a *Pseudomonas syringae* pv. tomato DC3000 effector, abolishes the interaction of GRP7 with the *FLS2* and *EFR* RNA by suppressing the RNA‐binding activity of GRP7. This suppression was found to correlate with a reduction in FLS2 protein levels. Whether HopU1‐mediated suppression of GRP7 has wider impacts on global alternative splicing and subsequent effects on PAMP‐Triggered Immunity remains an open question. (b) CAF1 may contribute to immunity by deadenylating a repressor of pathogenesis‐related (PR) proteins. The expression of *A. thaliana* CAF1a and AtCAF1b has been found to be induced upon multiple treatments such as stress‐related hormones and biotic stress. It has been proposed that upon elicitation of immunity, *CAF1a* and *CAF1b* are transcriptionally upregulated and participate in the deadenylation and thus degradation of a repressor of *PR* gene transcription (currently unknown). Consequently, in *A. thaliana* CAF1a and CAF1b overexpressing lines, the repressor is highly deadenylated and degraded, allowing high expression of *PR1* and *PR2* genes and increased resistance to *P. syringae* pv. tomato DC3000. The opposite is true for *caf1a* and *caf1b* mutants. (c, d) Dual mode of action of EIN2. Upon ethylene perception, the carboxyl domain of EIN2 (EIN2‐C) is cleaved and can play two different roles: (c) EIN2‐C can shuttle to the nucleus to stabilise two transcription factors (EIN3/EIL1) that positively regulate ethylene responses; (d) EBF1/2 promotes degradation of EIN3/EIL1 transcription factors. EIN2‐C can bind to the 3′UTR of *EBF1/2* transcripts in the cytoplasm and together with the nonsense‐mediated mRNA decay machinery re‐localise them to the processing bodies to promote translational repression. The translational repression of *EBF1/2* results in the expression of ethylene responsive genes. Created with BioRender.com.

Unicellular and multicellular plant pathogens can directly target components of the splicing machinery to disrupt host immunity as a virulence strategy. In *A. thaliana*, the RBP GLYCINE RICH PROTEIN 7 (GRP7) plays a critical role in PTI that is dependent upon its ability to bind RNA via its N‐terminal RRM domain (Figure 2). GRP7 belongs to the hnRNP class of splicing factors that modulate global AS (Streitner et al., 2012). During infection by *P. syringae* pv. tomato DC3000, the type III secretion system‐secreted effector HopU1 directly targets GRP7 and its functionally redundant paralogue GRP8 to suppress PTI (Fu et al., 2007). HopU1 is a mono‐ADP‐ribosyltransferase that ADP‐ribosylates arginine 49 within the RRM of GRP7. This modification abolishes the ability of GRP7 to interact with its mRNA targets, particularly *FLS2* and *EFR*, which correlates with a reduction in the accumulation of these immune receptors (Jeong, Lin, et al., 2011; Nicaise et al., 2013). However, how HopU1‐directed suppression of GRP7 affects the AS of its other RNA substrates during infection remains to be explored.

More recently, Li and Kou (2025) used a splicing reporter system developed by Huang et al. (2020) to screen for *Ralstonia solanacearum* effectors capable of affecting AS and found that the RipP2 effector acetylates the SWQDLKD motif of the RNA‐binding RMMH domain of the tomato splicing factor SR34a, thereby promoting intron splicing in an RLPK:LUC reporter construct, but suppressing splicing in four immune‐related genes (RBP, ER68, SNR and U2AF65C). While overexpression of *SR34a* enhanced resistance, co‐expression with functional RipP2 restored susceptibility, indicating that RipP2 interferes with the splicing of defence‐associated genes by SR34a. Splicing reporters such as the RLPK:LUC reporter, in which an alternatively spliced region of mRNA is fused to a luciferase (LUC) transcript, with the spliced transcript generating functional LUC protein and the intron‐retained transcript failing to produce LUC (Huang et al., 2020) represent an attractive option to monitor dynamic changes in AS during infection and immune challenge. However, appropriate controls must be performed to ensure that neither the reporter transcript nor the reporter protein influence immune responses, and as this example illustrates, the responses of splicing reporters may differ from that of individual genes.

The oomycetes *Phytophthora infestans* and *Phytophthora sojae*, the causal agents of late blight in solanaceous crops and root rot in soybean, respectively, also perturb AS during infection by targeting host RBPs (Gui et al., 2022; Huang et al., 2017, 2020; Sun et al., 2024). *Phytophthora sojae* deploys the virulence effector *Phytophthora* Suppressor of RNA silencing 1 (PSR1) to directly inhibit the activity of PSR1‐INTERACTING PROTEIN 1 (PINP1), which contributes to disease progression (Qiao et al., 2015). PINP1, also referred to as pre‐mRNA splicing factor 16, belongs to the MUT6 family of DEAD‐box RNA helicases that function in pre‐mRNA splicing and sRNA biogenesis. Suppression of the RNA‐binding activity of PINP1 either by overexpression of *PSR1* or RNAi silencing of *PINP1* in *A. thaliana* triggers AS changes in over 5000 genes, particularly intron retention. For example, an intron‐retained splice variant of *ALLENE OXIDE CYCLASE* (*AOC*), involved in JA biosynthesis, encodes an enzymatically inactive protein isoform that was stimulated in *PINP1*‐suppressed conditions. The canonical isoform of *AOC* could promote resistance to *Phytophthora* infection, whereas the intron‐retained isoform could not. Similar results were found for other genes associated with pathogen defence, demonstrating that *P. sojae* utilises PSR1 to alter global intron retention by targeting PINP1 as a virulence strategy (Gui et al., 2022).

Transcriptome‐wide analysis of AS in tomato leaves revealed that *P. infestans* can suppress the AS of positive regulators of the immune response, while enhancing the AS of negative regulators. To identify novel virulence effectors that manipulate host AS, Huang and colleagues developed a novel luminescence‐based reporter system to screen for effectors that regulate host splicing, termed splicing regulatory effectors (SRE). Amongst the identified proteins, SRE3 and SRE7 directly interact with the host core spliceosome U1‐70k subunit to manipulate host AS, which was proposed to be responsible for SRE3‐directed suppression of host immunity (Huang et al., 2020).

Plant‐parasitic nematodes also secrete effectors that interfere with host AS processes. The soybean cyst nematode *Heterodera glycines* and beet cyst nematode *Heterodera schachtii* produce a stylet‐secreted virulence effector named 30D08. 30D08 localises to the nucleus and interacts with the auxiliary spliceosomal component SMU2. Transgenic *A. thaliana* plants expressing 30D08 under the control of the SMU2 promoter displayed changes in AS that were hypothesised to contribute to pathogen survival (Verma et al., 2018).

The root knot nematode *Meloidogyne incognita* was found to secrete a virulence effector named MiEFF18 that interacts with SmD1, a core component in RNA splicing and quality control pathways that is also implicated in post‐transcriptional gene silencing (Elvira‐Matelot et al., 2016). Ectopic expression of MiEFF18 in *A. thaliana* roots disrupted AS and correlated with changes in AS observed in the *smd1b* mutant, which has reduced SmD1 functionality. MiEFF18‐directed remodelling of the host transcriptome may therefore contribute to the formation of the characteristic giant cells that are essential for parasitism (Mejias et al., 2021). Taken together, these studies highlight the importance of splicing as a mechanism to reprogramme the transcriptome during plant defence responses.

### RNA editing

RNA editing is a process that modifies RNA through insertions, deletions and substitutions to fine‐tune their function, translation and lifetime. Although RNA editing has been observed in the nucleus and cytosol, it mainly occurs in the mitochondria and plastids in plants (Hao et al., 2021; Knoop, 2023). Deamination of cytidine (C) to uridine (U) is the most frequent modification in plants and is carried out by the RNA editing complex, which includes both pentatricopeptide repeat (PPR) and RRM domain‐containing proteins, RNA editing factors/RNA editing interacting proteins and zinc‐finger proteins (Ichinose & Sugita, 2017; Lu, 2018).

RNA editing alters the RNA sequence and therefore potentially affects the characteristics of the transcript, including start and stop codon recognition, splicing, miRNA maturation and binding or changes to the sequence of the encoded protein (Rodrigues et al., 2017). Since RNA editing can add new properties to RNA and encoded proteins, it is expected that RNA editing factors are involved in plant immunity.

An example of an RNA editing factor involved in immunity is *A. thaliana* OVEREXPRESSOR OF CATIONIC PEROXIDASE 3 (OCP3). OCP3 is involved in RNA editing of chloroplast gene transcripts and in disease resistance by modulating the editing efficiency of plastid *ndhB* (García‐Andrade et al., 2013). The rice RRM domain protein OsRRM2 was also found to promote editing of *ndhB* and to contribute to resistance to *Magnaporthe oryzae* (Gu et al., 2024). Another example is SLOW GROWTH 2 (SLO2), an *A. thaliana* PPR protein involved in mitochondrial RNA editing, which is required for modulating the plant's response to biotic and abiotic stresses. *slo2* mutants showed increased susceptibility to the necrotrophic pathogen, *Botrytis cinerea* (Zhu et al., 2014).

Multiple Organellar RNA editing Factors (MORFs) are involved in C‐to‐U RNA editing in the mitochondria and chloroplast. *Nicotiana benthamiana* MORF8 was shown to negatively affect plant immunity; silencing of MORF8 was found to result in enhanced disease resistance to the oomycete pathogen *Phytophthora parasitica* (Yang et al., 2020). MORF genes were also observed to be downregulated during *Xanthomonas arboricola* pv. pruni (*Xap*) infection in peach (*Prunus persica*), which was speculated to modify stress responses (Zhang et al., 2021). A similar trend was observed in resistant kiwifruit upon *Pseudomonas syringae* pv. *actinidiae* infection, wherein MORF7 and MORF2.1 downregulation resulted in reduced RNA editing, which correlated with increased disease resistance (Xiong et al., 2022).

Many RNA editing RBPs in *A. thaliana* show altered RNA‐binding activity in response to immune elicitation with flg22, which indicates that they may play a role in PTI (Bach‐Pages, Chen, et al., 2020). Multiple RBPs involved in RNA editing, mostly in the chloroplast, were inhibited 12 h post flg22 treatment. These included proteins involved in C‐to‐U RNA editing and pseudouridine synthases, which agree with earlier studies showing that pathogen challenge inhibits chloroplastic RNA editing in plants (García‐Andrade et al., 2013). The biological significance of the inhibition of RNA editing upon immune stimulation deserves further investigation.

### Polyadenylation and m^6^A modification

After 3′ end definition during transcription termination, most eukaryotic mRNAs are modified at their 3′ end by addition of a poly(A) tail that protects them from degradation and aids translation (Eckmann et al., 2011). About 70% of *A. thaliana* genes can undergo alternative polyadenylation (APA), which alters the stability, translation or functionality of those genes by modifying the coding sequence and/or the length and properties of their 3′ untranslated region (UTR; Wu et al., 2011). APA is an important mechanism regulating gene expression in different organisms, including immune responses (Kondrashov et al., 2009).

One of the best studied RBPs involved in APA is *A. thaliana* FLOWERING TIME CONTROL PROTEIN (FPA), for which a mechanistic link to immunity is known. FPA regulates the 3′ mRNA polyadenylation of the transcriptional repressor ETHYLENE RESPONSE FACTOR 4 (ERF4) (Lyons et al., 2013). The PTI‐induced ROS burst is positively regulated by the canonical isoform of ERF4, but it is suppressed by the alternative isoform produced following APA, which lacks the ERF‐associated amphiphilic repression (EAR) motif. FPA partially inhibits the induction of APA isoforms of ERF4 by regulating 3′ end and polyadenylation site choice, consequently repressing the formation of EAR‐lacking ERF4 (Lyons et al., 2013). However, both EAR‐lacking ERF4 and FPA suppress ROS, indicating that FPA might contribute to the suppression of the ROS burst by regulating APA of other defence‐related genes (Lyons et al., 2013). In this scenario, PTI‐induced APA events are inhibited by FPA to restrict unnecessary resource allocation to defence mechanisms.

RNA can also be co‐transcriptionally modified via methylation, most notably through internal N^6^‐methyladenosine (m^6^A) modifications, which affect RNA stability and turnover (Chen et al., 2024; Luo et al., 2014; Martínez‐Pérez et al., 2017). m^6^A modifications are introduced by m^6^A methyltransferases, or writers, and removed by demethylases or erasers. Global m^6^A profiling of *A. thaliana* following the induction of PTI revealed dynamic changes in m^6^A modification and in the interaction of m^6^A sites with the m^6^A reader, EVOLUTIONARILY CONSERVED C‐TERMINAL REGION2 (ECT2) (Chen et al., 2024). This suggests a role for m^6^A modifications in stabilising the overall transcriptome, while facilitating turnover of PTI‐induced mRNA. Consistent with this, polysome profiling of PTI‐induced plants suggested that m^6^A enhances immune‐associated translation, while m^6^A was also found to target SA‐induced transcripts for sequestration in cytosolic condensates by the reader ECT1, thereby dampening the immune response (Chen et al., 2024; Lee et al., 2024).

Plants impaired in m^6^A modification, including plants with DEX‐induced silencing of the major methyltransferase MTA, showed increased susceptibility to biotrophic pathogens. However, an earlier study by Prall et al. (2023) found m^6^A‐deficient plants to be more resistant to bacterial and fungal infections. Similarly, Furci et al. (2024) found that infection of *A. thaliana* with *Hyaloperonospora arabidopsidis* caused a global reduction in m^6^A, while an m^6^A deficient mutant showed enhanced resistance. This suggests that the pleiotropic effects of m^6^A modifications on plant processes can affect mutant phenotypes.

Epitranscriptomic modifications associated with immunity are not limited to methylation. For example, Lu et al. (2024) recently showed that in rice infected with *M. oryzae*, increased expression of the ac4C writer OsNAT10/OsACYR (N‐ACETYLTRANSFERASE FOR CYTIDINE IN RNA), which catalyses N^4^‐acetylcytidine (ac4C) modification of mRNA, promotes translation of immune‐related transcripts to facilitate rapid activation of immune responses.

### Nuclear RNA export and import

When mRNAs are fully processed, they engage with export factors that enable them to traverse through nuclear pore complexes (NPCs). Export rates influence the number of mRNAs available for translation and the time an mRNA spends in the cytoplasm, controlling many biological processes, including stress responses (Van Ruyskensvelde et al., 2018). Intriguingly, one recent study of mammalian immune responses posited that rather than controlling mRNA expression, nuclear export rates of immune‐related genes complement mRNA decay, such that highly responsive mRNAs are exported efficiently, but show a correspondingly high rate of mRNA degradation (Lefaudeux et al., 2022). It remains to be shown whether this applies in plants.

A number of RBPs implicated in mRNA export and/or RNA import play a role in plant immunity, including SDE5, MOS11, NUP96, NUP160, SEH1 and HRP1 (Dufu et al., 2010; Germain et al., 2010; Hernandez‐Pinzon et al., 2007; Pan et al., 2012; Sugiura et al., 2007; Uddin et al., 2017; Wiermer et al., 2012; Xu et al., 2014; Zhang et al., 2005). For example, mutants of the nuclear‐localised protein MOS11 accumulate more mRNA in the nucleus and partially suppress the enhanced disease resistance phenotype of *snc1*, which carries a gain‐of‐function mutation in a TIR‐NB‐LRR type *R* gene (Germain et al., 2010). MOS11 interacts with the DEAD‐box RNA helicase UAP56, a component of the transcription and export complex, which acts to recruit the mRNA export receptor to the NPC, resulting in nucleocytosolic translocation of mRNA and has also been implicated in mRNA export under abiotic stress (Rödel et al., 2024).

*SILENCING DEFECTIVE 5* (*SDE5*) encodes an RBP involved in transgene silencing and production of ta‐siRNAs, which shows sequence similarity to the human export factor TAP (Hernandez‐Pinzon et al., 2007). Current evidence suggests that SDE5 acts together with Argonaute AGO1 in recruiting RNA‐dependent RNA polymerase 6 to RNAs that have been targeted for silencing and may also have functions in nuclear RNA export or import (Uddin et al., 2017; Yoshikawa et al., 2021). SDE5 contributes to ETI and suppresses PTI, having a positive effect on SA‐mediated defences, and a negative effect on JA‐mediated processes, suggesting that it participates in interactions between these signalling pathways (Uddin et al., 2017). Accordingly, SDE5 contributes to increased resistance to the biotrophic pathogen *P. syringae* pv. tomato DC3000 and increased susceptibility to the necrotrophic bacterium *Pectobacterium carotovorum* subsp. *carotovorum* (formerly *Erwinia carotovora*; Uddin et al., 2017).

### RNA stability/decay

The pool of a given RNA that is available for translation depends not only on its synthesis, processing and nuclear export rates but also on its lifetime. Hence, transcript stability, which depends on the rate of degradation, largely determines the abundance of RNA that is available to be translated. mRNA stability can be globally or specifically regulated to quickly adjust to different cellular states and in response to different environmental conditions, including biotic stresses (Jiao et al., 2008; Yu et al., 2019).

#### Deadenylation

mRNA needs to be deadenylated (shortening/removing the poly(A) tail) before it can either be degraded in a 3′–5′ direction or be decapped and degraded in the 5′–3′ direction (Garneau et al., 2007). There are multiple deadenylase complexes in plants, such as the poly(A) ribonuclease PARN, the poly(A) nuclease PAN and the CCR4/CAF1 deadenylase complex. The PARN and the CCR4/CAF1 deadenylase complex have both been linked to plant immunity (Johnson et al., 2018; Liang et al., 2009; Walley et al., 2007, 2010).

For example, the expression of *A. thaliana* CCR4‐ASSOCIATED FACTOR 1a and b (AtCAF1a and AtCAF1b) is induced upon multiple treatments such as stress‐related hormones (JA, SA and abscisic acid) and biotic stress (Figure 2; Liang et al., 2009; Walley et al., 2007, 2010). It has been speculated that upon elicitation of immunity, *CAF1a* and *CAF1b* are transcriptionally upregulated and participate in the deadenylation, and thus degradation, of a hypothetical repressor of *PR* gene transcription (Liang et al., 2009). Consequently, in *A. thaliana* CAF1a and CAF1b overexpressing lines, the repressor is highly deadenylated and degraded, allowing high expression of *PR1* and *PR2* genes and increased resistance to *P. syringae* pv. tomato DC3000, and the opposite is true for *caf1a* and *caf1b* mutants (Liang et al., 2009). Similarly, overexpression of CaCAF1a in tomato (*Solanum lycopersicum*) has been reported to lead to increased resistance against *P. infestans* (Sarowar et al., 2007). In agreement, virus‐induced gene silencing of CaCAF1a in pepper (*Capsicum annum*) results in increased susceptibility to the bacterial pathogen *Xanthomonas axonopodis* pv. *vesicatoria* (Sarowar et al., 2007).

#### Decapping

After deadenylation, RNA is decapped to enable 5′–3′ degradation. Decapping involves removal of the m^7^G cap at the 5′ end of the mRNA and is mainly carried out by the decapping enzyme DECAPPING2 (DCP2), the decapping activators DCP1/5, VARICOSE and PROTEIN ASSOCIATED WITH TOPOISOMERASE1 (PAT1), and EXORIBONUCLEASE 4 (XRN4), which degrades the RNA. PTI induces mRNA decapping to inhibit general translation (Wang, Wang, et al., 2022; Wang, Zhang, et al., 2022); many of the members of the decapping complex are implicated in plant immunity.

The DCP2 enzyme functions together with the co‐activator DCP1 (and other factors) in a decapping complex and has been recently described to be involved in immunity‐related mRNA decay in the P‐bodies (Yu et al., 2019). Upon flg22 perception, DCP1 is phosphorylated by two MAPKs (MPK3/6), which promotes dissociation from DCP2 and association with XRN4 (Yu et al., 2019). DCP1‐XRN4 stimulates XRN4 exonuclease activity or XRN4 access to mRNAs, thus resulting in the degradation of certain decapped RNAs and contributing to the downregulation of a subset of genes during the onset of PTI (Yu et al., 2019). *DCP1* and *DCP2* silencing leads to decreased PTI‐induced defence gene expression and increased susceptibility to *P. syringae*, which may be due to reduced degradation of genes that negatively regulate PTI and/or changes in resource allocation linked with changes in mRNA decay.

PAT1 is a decapping enhancer that, together with LSM1‐7, links deadenylation and decapping by binding the 3′ end of deadenylated mRNAs and promoting decapping of specific transcripts (Tharun, 2009). PAT1 also functions in translational inhibition and P‐body formation (Roux et al., 2015). PAT1 is post‐transcriptionally regulated in response to flg22, which promotes its phosphorylation by MPK4 (and to a lesser extent MPK6) and its re‐localisation to P‐bodies (Roux et al., 2015). *pat1* mutants exhibit autoimmunity, display increased resistance to *P. syringae* pv. tomato DC3000, and high constitutive expression of *PR1* and *PR2* genes (Roux et al., 2015). Although PAT1 provides an example of a mechanism whereby MPKs regulate mRNA decay machinery during immune responses, the downstream RNAs regulated by PAT1 remain unknown. However, as LSM1‐7 interacts with different stress‐responsive transcripts depending on the source of stress (Perea‐Resa et al., 2016) it seems probable that PAT1 also contributes to stress‐specific mRNA decay.

Additional support for the contribution of decapping to susceptibility comes from the discovery that the Nudix hydrolase effector AvrM14 from the flax rust fungus *Melampsora lini* is specifically involved in decapping of host mRNA transcripts as a novel virulence strategy to interfere with plant immunity (McCombe et al., 2023).

#### Other RNA stability/turnover mechanisms

In addition to decapping, a number of RBPs involved in other aspects of RNA stability/turnover have been described to be involved in immunity. For example, PVPRP7 MRNA‐BINDING PROTEIN (PRP‐BP) is a cytoplasmic RBP that binds specifically to the 3′UTR of *PvPRP1*, which encodes a cell wall‐associated proline‐rich protein. The RNA‐binding activity of PRP‐BP is increased upon treatment of bean (*Phaseolus vulgaris*) cells with elicitors from *Colletotrichum lindemuthianum*, leading to destabilisation and downregulation of *PvPRP1* (Sheng et al., 1991; Zhang et al., 1993; Zhang & Mehdy, 1994). Interestingly, the RNA‐binding activity of PRP‐BP was found to be regulated by the redox state of the sulphhydryl groups. This suggests that the RNA‐binding activity of PRP‐BP is modulated by the redox changes that typically occur after pathogen infection (Zhang & Mehdy, 1994). Hence, upon elicitor treatment, PRP‐BP is post‐translationally activated and binds to the target *PvPRP1* mRNA to promote its degradation.

The rice cytoplasmic Tetratricopeptide repeat‐containing protein BROAD‐SPECTRUM RESISTANCE KITAAKE‐1 negatively regulates plant immunity by binding to multiple defence‐related *OsPAL* (phenylalanine ammonia‐lyase) transcripts (*OsPAL1‐7*) and promoting their turnover (Zhou et al., 2018). Accordingly, *bsk‐k1* mutants accumulate higher *OsPAL* transcripts and show enhanced resistance to different races of the fungus *M. oryzae* and the bacterium *Xanthomonas oryzae* pv. *oryzae* (Zhou et al., 2018).

Rice bZIP TF AVRPIZ‐T‐INTERACTING PROTEIN 5 (APIP5) acts as both a nuclear transcription factor and a cytoplasmic RBP and negatively regulates programmed cell fate and blast resistance by regulating the turnover of mRNAs that include the cell death‐ and defence‐related genes OsLSD1 and OsRac1. Interestingly, APIP5 has been reported to be targeted by the effector AvrPiz‐t of the blast fungus *M. oryzae* (Zhang et al., 2022).

PR‐10 proteins from diverse species have been described to be ribonucleases and to have important roles against multiple species of bacteria and fungi. For example, CaPR‐10 is a ribonuclease from pepper (*Capsicum annuum*) that has been shown to play a role in defence against multiple pathogens including *Xanthomonas campestris* pv. *vesicatoria*, *Phytophthora capsici* and tobacco mosaic virus (Park et al., 2004). CaPR‐10 was shown to interact with LEUCINE‐RICH REPEAT 1 protein, leading to HR‐like cell death and activation of defence signalling (Choi et al., 2012). Silencing of PR‐10 in pepper results in increased susceptibility to *X. campestris*, whereas overexpression in *A. thaliana* leads to increased resistance to *P. syringae* pv. tomato DC3000 and *H. arabidopsidis* (Choi et al., 2012).

PR‐10 proteins from several other species have also been implicated in plant immunity (Chadha & Das, 2006; Liu & Ekramoddoullah, 2006; Pungartnik et al., 2009; Zhou et al., 2002). Likewise, PR‐4 proteins have also been shown to possess ribonuclease activity and to be involved in immunity (Filipenko et al., 2013). However, a recent paper raises questions about the controls used to confirm ribonuclease activity, suggesting that in some instances activity could be linked to co‐purifying proteins (Longsaward et al., 2023). Further validation may be needed to confirm whether the effects of these proteins on immunity are indeed linked to ribonuclease activity.

#### mRNA surveillance pathways

Eukaryotic cells possess a range of mRNA surveillance mechanisms to ensure the quality of cellular mRNAs and encoded proteins. These include nonsense‐mediated mRNA decay (NMD), nonstop mRNA decay (NSD) and no‐go mRNA decay (NGD) (Doma & Parker, 2007). The best‐studied pathway for mRNA surveillance is NMD, which targets mRNAs with aberrant translation termination and involves UPF1/2/3, SMG1/7 and the exon‐junction complex. NMD has been extensively linked to immunity against bacteria and viruses in plants (Garcia et al., 2014; Ohtani & Wachter, 2019; Shaul, 2015) and mutations in NMD proteins such as UPF1, UPF5 and SMG7 lead to increased resistance to *P. syringae* pv. tomato DC3000. Moreover, NMD mutants have autoimmune phenotypes and display high constitutive *PR* gene expression and increased SA content (Jeong, Kim, et al., 2011; Rayson et al., 2012; Riehs‐Kearnan et al., 2012; Shi et al., 2012). It has also been reported that NMD controls the turnover of disease resistance (R) genes since the transcripts of TIR‐NBS‐LRR (TNL)‐ and CC‐NBS‐LRR (CNL)‐ immune receptors displayed an increased half‐life in *smg7* mutants (Gloggnitzer et al., 2014; Jung et al., 2020).

Interestingly, upon infection with *P. syringae* pv. tomato, NMD activity is reduced, but the mRNA levels of UPF1 and UPF3 are upregulated (Jeong, Kim, et al., 2011), while UPF1 and UPF3 proteins show increased degradation (Jung et al., 2020). Degradation of UPF1 and UPF3 is also observed in response to flg22, although NMD activity recovers more quickly in response to PTI than pathogen inoculation, which suggests that *P. syringae* effectors are acting to suppress NMD (Jung et al., 2020). Collectively, this suggests that repression of NMD contributes to the stabilisation of gene expression for genes with important roles in the defence response, but may also benefit the pathogen.

### Stress granules and P‐bodies

SGs and P‐bodies are membraneless organelles. They are formed by the condensation of mostly translationally inactive mRNAs along with RBPs; although there is evidence of some SG‐localised transcripts undergoing translation (Mateju et al., 2020). These structures decrease the available pool of translating RNA and are involved in post‐transcriptional regulation and translational control. SGs have been observed in the cytosol and in chloroplasts and are present in cells regardless of stress conditions, but their composition and dynamics change under stress (Aerts et al., 2022; Kearly et al., 2022; Youn et al., 2019).

During stress conditions, including biotic stress, mRNAs can be re‐localised to SGs and P‐bodies, where they are either stored or degraded (Chantarachot & Bailey‐Serres, 2018; Weber et al., 2008). Many processes involved in mRNA turnover are thought to occur in P‐bodies, due to the presence of proteins involved in deadenylation, decapping, miRNA‐targeted gene silencing and NMD. However, it has also been reported that mRNAs can be stored in P‐bodies without undergoing degradation (Dave & Chao, 2020; Horvathova et al., 2017; Hubstenberger et al., 2017; Li et al., 2015; Merchante et al., 2015).

Recent studies have shown that there is a potential link between immune responses and the re‐localisation of RNAs to P‐bodies and SGs (Li et al., 2015; Maldonado‐Bonilla et al., 2014; Merchante et al., 2015; Petre et al., 2016; Roux et al., 2015; Yu et al., 2019). P‐bodies have been shown to disassemble and reassemble rapidly upon pathogen perception, which indicates that the fates of the mRNAs stored in the P‐bodies dynamically change during immune responses (Yu et al., 2019). It has been suggested that P‐bodies mediate degradation of transcripts encoding negative regulators of immunity in plants to enhance immune responses (Yu et al., 2019). Additionally, there is evidence that some pathogens target P‐bodies to interfere with RNA metabolism and disrupt defence responses (Petre et al., 2016) as has been observed in other organisms, such as mammals (Ariumi et al., 2011; Pérez‐Vilaró et al., 2015). Flg22 treatment stimulates the RNA‐binding activity of several proteins associated with P‐bodies, including PAT1, PAT1H1, LSM6A/B, ETHYLENE INSENSITIVE 2 (EIN2) and RH6 (Bach‐Pages et al., 2020), which further indicates that P‐bodies play a role during defence responses.

EIN2 is a protein associated with P‐bodies and SGs that has been described to play a role in responses to various stimuli, including biotic stress (Gazzarrini & Mccourt, 2003; Lu et al., 2022; Qiao et al., 2012; Rin et al., 2017; Salvador‐Guirao et al., 2018; Zhang et al., 2020). EIN2 is an evolutionarily conserved ethylene‐signalling component that contains a cytoplasmic domain and a transmembrane domain anchored to the ER (Ju et al., 2015). Ethylene signalling is an integral part of PTI (Alonso et al., 1999) and ethylene perception results in the cleavage of the carboxyl terminus of EIN2. This cleaved cytoplasmic domain called EIN2‐CEND (EIN2‐C) is translocated into the nucleus where it stabilises ETHYLENE INSENSITIVE 3 (EIN3) and EIN3‐like (EIL1), transcription factors that positively regulate ethylene responses (Ju et al., 2015; Qiao et al., 2012; Wen et al., 2012). EIN2‐C was also observed to be present in the cytoplasm, where it binds to the 3′UTR of EIN3 BINDING F‐Box 1/2 (EBF1/2) mRNAs and promotes their translational repression and localisation to P‐bodies (Li et al., 2015; Merchante et al., 2015) (Figure 2). EBF1 and EBF2 regulate the protein levels of EIN3/EIL1 and promote their degradation. Therefore, through translational repression of EBF1/2, EIN2 promotes the expression of ethylene‐responsive genes.

There has been some speculation on whether EIN2 directly binds to *EBF1/2* mRNAs as EIN2 lacks distinct RBDs. However, EIN2 has been confirmed to interact with mRNA *in vivo*, and the RNA‐binding activity of EIN2 increases within 2 h after PTI elicitation (Bach‐Pages et al., 2020). In rice, a glycine‐tyrosine‐phenylalanine (GYF) domain‐containing protein MHZ9 has been identified which interacts with OsEIN2‐C in the P‐body. MHZ9 was also observed to directly bind to *OsEBF1/2* mRNAs for their translational repression (Huang et al., 2023). Hence, EIN2 could be a part of a complex that interacts with mRNAs to promote their re‐localisation to P‐bodies.

Several independent groups have reported that *ein2* mutants have reduced immunity (Boutrot et al., 2010; Mersmann et al., 2010; Tintor et al., 2013). Consistently, *ein2* mutant plants are more susceptible to *P. syringae* pv. tomato DC3000 infection (Clay et al., 2009; Mersmann et al., 2010; Tintor et al., 2013; Washington et al., 2016). It was also observed that flg22‐induced phosphorylation of MPK3 and MPK6 was inhibited in *ein2* mutants (Wang et al., 2023). However, some contradictory results have been reported with regard to the involvement of EIN2 in callose deposition and MAPK activation (Mersmann et al., 2010). Comprehensive identification and characterisation of mutants involved in ethylene perception and signalling is required to draw solid conclusions.

Recently, plant TANDEM CCCH ZINC‐FINGER PROTEINS (TZFs) such as TZF9 and GhZFP1 have been associated with P‐bodies and SGs (Guo et al., 2009; Maldonado‐Bonilla et al., 2014). TZFs typically contain an arginine‐rich (RR) region followed by two CCCH‐type zinc‐finger motifs arranged in tandem (Bogamuwa & Jang, 2014). In *A. thaliana*, TZF9 localises to P‐bodies and plays a role in post‐transcriptional regulation of PTI responses. *tzf9* mutants have altered immune responses and are more susceptible to *P. syringae* pv. tomato DC3000. Identification of mRNAs regulated by TZF9 could explain the mechanism by which it modulates plant immunity (Maldonado‐Bonilla et al., 2014).

### Ribosomes and translation

Plants can adapt to stress and immune challenges by regulating mRNA translation, which allows rapid control of protein abundance (Xu et al., 2017; Guo, 2018; Son & Park, 2023, for review) and as previously noted, RNA modifications including capping, editing, APA and methylation and other epitranscriptomic modifications can all alter translation efficiency. Traditionally, ribosomes were seen as homogenous macromolecules involved in protein synthesis. However, recent findings have led to the identification of ‘specialised ribosomes’, with heterogeneous composition of ribosomal RNAs and proteins or different post‐translational modifications (PTMs) of the ribosomal components (Mauro & Edelman, 2002; Xue & Barna, 2012). These heterogeneous ribosomes can preferentially translate different subsets of mRNAs (Guo, 2018). Ribosomes can be located in the cytosol, endoplasmic reticulum or inside mitochondria and chloroplasts and have been described to vary in response to different factors such as cellular status, environmental conditions or developmental stage (Venezia et al., 2019).

Several recent studies have reported that translation can be selectively regulated during plant immune responses (Meteignier et al., 2017; Wang, Wang, et al., 2022; Wang, Zhang, et al., 2022; Xu et al., 2017; Yoo et al., 2020), and several ribosomal proteins and translation‐related proteins have been seen to be post‐translationally altered or to show altered RNA‐binding activity in plants treated with immune elicitors (Bach‐Pages, Chen, et al., 2020; Eskelin et al., 2019; Fakih et al., 2016; Siodmak et al., 2023). Therefore, it is clear that the composition of ribosomes and the proteins associated with them is dynamically regulated during stress and pathogen responses. This is expected to result in distinct translation programmes that regulate plant responses to different environmental cues, including biotic stresses.

Targeting of plant ribosome biogenesis by pathogen effectors has been found to be important for pathogenicity. For example, the *P. infestans* RNA‐binding effector Pi23226 interferes with host ribosome biogenesis by binding to the 3' end of 25S rRNA precursors and inhibiting global protein translation (Lee et al., 2023). Moreover, *Blumeria graminis* effector CSEP0064/BEC1054 binds to host ribosomes, thereby inhibiting the action of plant ribosome‐inactivating proteins that would otherwise lead to host cell death (Pennington et al., 2019).

## FUTURE OUTLOOK

**Figure 3:**
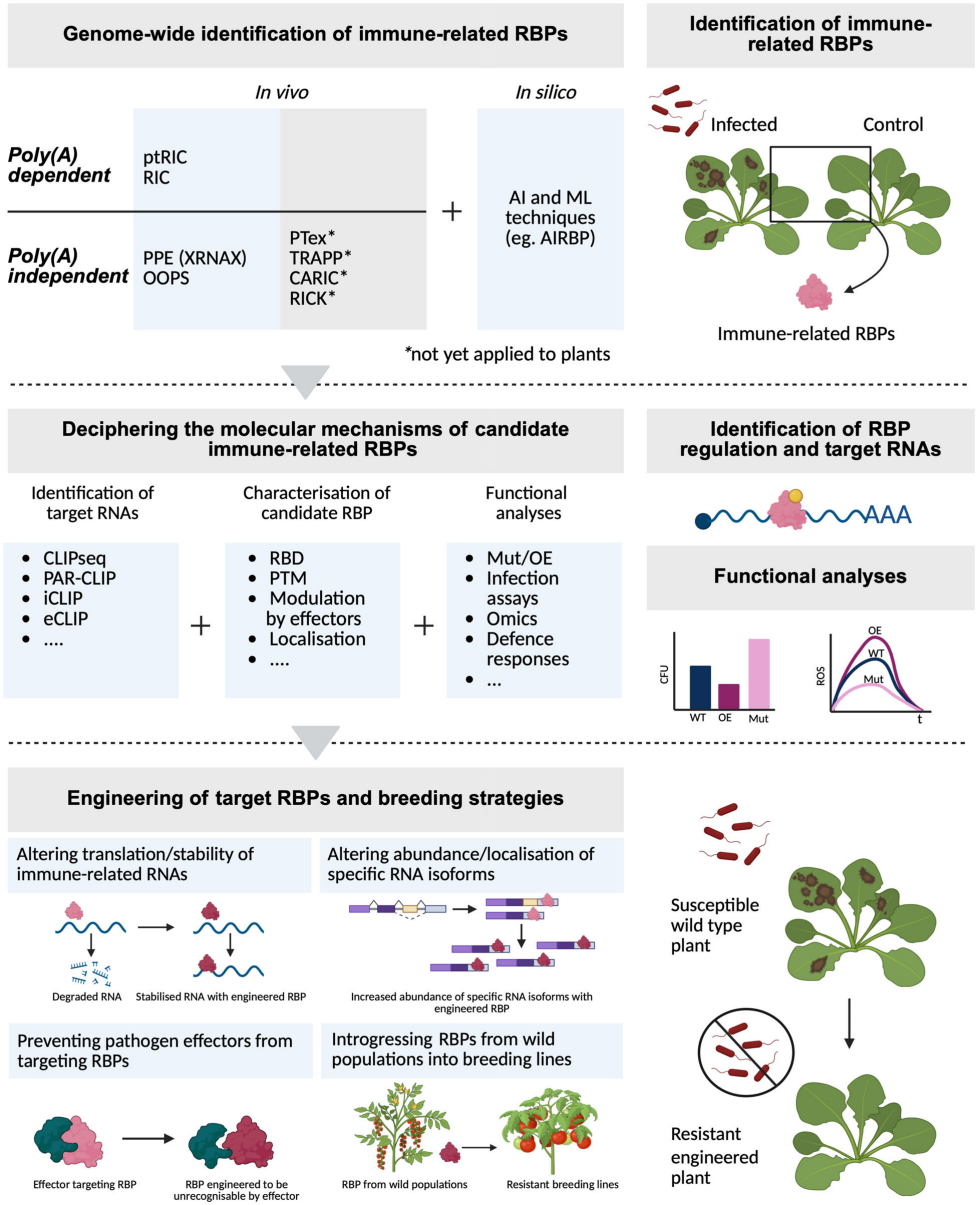
Use of genome‐wide techniques to identify immune‐related RNA‐binding proteins (RBPs) for breeding strategies. A range of techniques can be applied to identify RBPs on a genome‐wide scale, complemented by *in silico* approaches. RBPs that show differential activity during plant immune responses can be uncovered by comparing RBP activity in infected or immune‐challenged versus control tissue. CLIP‐seq and other techniques can identify the RNAs regulated by candidate RBPs. In‐depth characterisation of candidate RBPs can confirm their ability to bind RNA, reveal their RNA‐binding domains, post‐translational modifications regulating their activity, potential targeting and modulation by pathogen effector proteins, cellular localisation and other features. Functional analyses involving plants overexpressing or carrying mutations in RBPs (Mut/OE), infection assays, evaluation of defence responses and omics analyses (including splicing and alternative splicing) can help to elucidate the role of RBPs in plant immune responses. Finally, information gathered can inform the engineering of RBPs or target mRNAs and guide accelerated breeding and gene‐editing strategies aimed at enhancing resistance against pathogens; for example: (1) altering the translation or stability of immune‐related RNAs, (2) altering the abundance or localisation of specific RNA isoforms, (3) preventing RBPs from being targeted as susceptibility factors by pathogen effectors or (4) introgressing or engineering RBPs or RBP variants with the potential to enhance immune responses into breeding lines. *Not yet applied to plants. Created with BioRender.com.

Here we have summarised the increasing evidence for the critical role of RBPs in orchestrating the post‐transcriptional changes that occur during plant immune responses. This rapidly emerging field is currently benefiting from rapid advances in the approaches used to identify RBPs at a proteome‐wide scale, which will transform our knowledge about their scope and roles in plants (Figure 3).

In this context, RIC and derivatives have enabled the discovery of numerous RBPs previously unknown across various species (Bach‐Pages et al., 2020; Hentze et al., 2018). However, a limitation of ptRIC is that it relies on oligo(dT) capture, thereby excluding RNAs that lack poly(A) tails, including many small and some organellar RNAs. Recently, protocols that do not rely on oligo(dT) capture have been applied for the first time in plants. These include plant phase extraction (Zhang et al., 2023) and OOPS (Liu et al., 2020). Other recent techniques that have been developed for other organisms include phenol‐toluol extraction (PTex; Urdaneta et al., 2019), total RNA‐associated protein purification (TRAPP; Shchepachev et al., 2019), click chemistry‐assisted RIC (CARIC; Huang et al., 2018a, 2018b) and RIC using click chemistry (RICK; Bao et al., 2018), expanding the repertory of methodologies potentially available to study RBPs in plants. Additionally, experimental approaches can be complemented by improvements in protein structure prediction and *in silico* methods such as AIRBP, enabling identification of candidate RBPs using machine learning (Mishra et al., 2021). Although the efficiency of RBP isolation and identification varies amongst different methods, collectively they have the potential to identify RBPs interacting with non‐polyadenylated RNAs and to complement ptRIC to provide a comprehensive RBP census. Furthermore, as with other 'omic technologies, these methods provide an exciting opportunity to study the dual RBPomes of host and pathogen, including the extracellular RBPome at the interface between host and pathogen (Wang et al., 2024).

Although a wide range of RBPs have been linked to immunity, for many RBPs their precise role in RNA metabolism and/or immunity remains unknown. For example, a recent study by Li et al. (2024) identified a nuclear‐localised RBP targeted by the *P. infestans* effector Pi23014, which contributes to immunity in an RRM‐dependent manner, but the function of this RBP is unknown (Li et al., 2024). Because RBPs act on RNAs or are regulated by RNAs, it becomes essential to identify their target RNAs. A range of approaches building on crosslinking, immunoprecipitation and sequencing (CLIP‐seq) have been developed to identify RNAs bound by RBPs (Haroon et al., 2022; Lewinski et al., 2024; Mateos & Staiger, 2023). Application of these techniques to RBPs enables the identification of both target RNAs and the exact position(s) where binding occurs. Researchers have also begun to explore the potential of fusing or directing RNA base editors to RBPs to identify modified target RNAs, which can enable profiling of RBP‐RNA interactions with lower amounts of input material (Liang et al., 2024; Medina‐Munoz et al., 2024), complementing advances in single‐cell transcriptomic, epigenomic and spatial transcriptomic methodologies (Nobori et al. 2025). This data will be critical to decipher the role of RBPs in immunity.

RBP activity can be regulated by PTMs, and indeed, RBDs are enriched in PTM sites (Castello et al., 2016; Arif et al., 2018; England et al., 2022; Xu et al., 2022). Analyses of changes in RBP activity following immune elicitation have provided evidence that early changes may, at least partly, be controlled by PTMs (Bach‐Pages, Chen, et al., 2020; Roux et al., 2015; Yu et al., 2019). Similarly, a study by Sharma and colleagues indicates that multiple immunity‐linked RBPs, including the spliceosome‐associated RBPs MOS2 and MOS4, may be subject to regulation by SUMOylation (Sharma et al., 2021), consistent with the widely established role of SUMOylation in regulation of RNA processing and metabolism (Richard et al., 2017). Hence, understanding how the function of RBPs is modulated by PTMs is critical to understand the RBP‐mediated regulation that occurs during plant immune responses.

When considering the evidence for a specific role for RBPs in immunity, it is important to note that many RBPs have been identified as potential regulatory or susceptibility factors based on mutant or gene‐silencing phenotypes. However, since many RBPs have broad and pleiotropic functions, it is not unexpected that mutant or silenced lines display altered immune responses against pathogens, along with other phenotypic changes. In such cases, it may be difficult to disentangle specific roles of RBPs in immunity from their general functions in cellular processes. Evidence of immune‐elicited PTMs, along with changes in RNA‐binding activity, localisation or effector interactions may be useful contextual indicators of specific links to immunity.

While techniques such as ptRIC have opened a new window on protein–RNA interactions, uncovering interactions that were not previously known to exist, some of the proteins identified using these approaches have well‐established roles in cellular metabolism that do not relate to RNA biology. Such multifunctional proteins, commonly referred to as moonlighting proteins in cases where proteins have different independent functions, may contribute to immune processes in an RNA‐dependent or RNA‐independent manner (Castello et al., 2015; Curtis & Jeffery, 2021). For example, the metabolic enzyme glyceraldehyde‐3‐phosphate dehydrogenase (GAPDH) is a well‐established RBP in humans, which has recently been implicated in retrograde signalling processes in plants through regulatory interactions with the 5′UTR of transcripts of stress‐associated proteins (Moore et al., 2022). GAPDH isoforms have also been linked to plant immunity, with knockout mutants showing increased disease resistance, associated with increased ROS and constitutive autophagy (Henry et al., 2015). Therefore, it is important to identify the precise function of RBPs in cellular metabolism, as well as in plant immunity, and to investigate whether RBPs contribute to immunity both by regulating specific steps of the RNA lifecycle or through roles in other cellular processes.

We have highlighted the central role of RBPs in regulating cellular homeostasis both in steady‐state and in response to environmental, physiological and pathological stimuli. Since RBPs are critical for plant growth, development and survival, they represent potential targets for breeding programmes to improve plant traits, including pathogen resistance. Global analyses of the RBPome have the potential to complement other 'omic approaches, such as genomics, transcriptomics, metabolomics and proteomics in supporting a multi‐omic approach to accelerated plant breeding (Mahmood et al., 2022), while knowledge of the regulation, specificity, structure and function of RBPs can be used to engineer plants to enhance immunity. This could include modification of RNA‐binding affinity, specificity or modifications to enable RBPs to avoid being targeted by pathogen effectors (Figure 3). Finally, generating information regarding the RNAs that are bound or modified by RBPs could enable us to develop new strategies to achieve durable plant resistance to pathogens, for example by selectively expressing specific splice variants to change the abundance of different protein isoforms.

In conclusion, this review underscores the pivotal role of RBPs as key orchestrators of transcriptional changes during plant immune responses. While challenges remain in deciphering the precise functions of RBPs and understanding their impact on cellular processes, including immunity (Box 2), the multifaceted nature of RBPs presents promising avenues for enhancing plant resistance against pathogens through targeted breeding programmes and engineering approaches. By harnessing knowledge of RBP regulation, specificity and function, we can pave the way for new strategies for developing resilient crops with improved traits to support sustainable agricultural practices.

Box 2 **Open questions**

- Many RNA‐binding proteins (RBPs) play global roles in RNA biology and therefore interact with thousands of transcripts, but have been linked to plant immunity through studies focusing on individual, immune‐related transcripts. What is the broader impact of RBP regulation by host and pathogen on plant processes and plant immunity?
- What is the role of protein–RNA interactions in the non‐canonical and potentially moonlighting RBPs identified by empirical protein–RNA interaction assays such as RNA interactome capture?
- RBP activity is rapidly and dynamically altered following induction of plant immunity. What processes direct the post‐translational regulation of RBP activity following immune activation?
- Do differences in alternative splicing and protein isoforms between resistant and susceptible plants contribute to differences in disease resistance and can this be exploited to increase disease resistance?
- What processes underpin changes in organellar, and particularly chloroplastic RBP activity during plant immune responses; and how do these changes affect the central role of the chloroplast in plant immune responses?

## CONFLICT OF INTEREST

The authors declare no competing interests.

## Supporting information


**Table S1.** Representative RNA‐binding proteins involved in plant immunity. RBPs that have been linked to plant immunity, classified according to RNA metabolic pathway. Abbreviations: NMD, nonsense‐mediated decay; PBs, processing bodies; SGs, stress granules.

## Data Availability

Data sharing not applicable to this article as no datasets were generated or analysed during the current study.
